# 25-hydroxyvitamin D deficiency is associated with an increased risk of metabolic syndrome in patients with non-diabetic chronic kidney disease 

**DOI:** 10.5414/CN107498

**Published:** 2012-07-12

**Authors:** Stephanie Seiki, Michel Chonchol, Alfred K. Cheung, James S. Kaufman, Tom Greene, William L. Roberts, Gerard Smits, Jessica Kendrick, the HOST Investigators

**Affiliations:** 1 Division of Renal Diseases and Hypertension, University of Colorado School of Medicine, Aurora, CO,; 2 Renal Section, Medical Service, Veterans Affairs Salt Lake City Healthcare System,; 3 Division of Nephrology & Hypertension, University of Utah, Salt Lake City, UT,; 4 Renal Section, Medical Service, Veterans Affairs Boston Healthcare System and Boston University School of Medicine, Boston, MA,; 5 Research Service, Veterans Affairs Salt Lake City Healthcare System,; 6 Division of Epidemiology,; 7 Department of Pathology, University of Utah, Salt Lake City, UT and; 8 Denver Health Medical Center, Denver, CO, USA

**Keywords:** 25-hydroxyvitamin D, chronic kidney disease, metabolic syndrome

## Abstract

Background: Patients with chronic kidney disease (CKD) not requiring dialysis have a high prevalence of 25-hydroxyvitamin D (25(OH)D) deficiency but the relationship between 25(OH)D levels and metabolic syndrome is unknown in this population. Methods: This study analyzed stored plasma samples from 495 non-diabetic subjects with severe kidney disease, not yet on dialysis, who participated in the homocysteine in kidney and end stage renal disease study. Metabolic syndrome was defined as the presence of all three of the following: (1) Serum triglycerides ≥ 150 mg/dl or drug treatment for hypertriglyceridemia; (2) serum high density lipoprotein-cholesterol (HDL-C) < 50 mg/dl for women or < 40 mg/dl for men or drug treatment for dyslipidemia; and (3) blood pressure ≥ 130/85 mmHg or drug treatment for hypertension. Multivariate logistic regression models were used to evaluate the cross-sectional association between plasma 25(OH)D levels and metabolic syndrome. Results: The prevalence of metabolic syndrome increased as 25(OH)D levels declined, with the highest prevalence in participants with 25(OH)D levels < 20 ng/ml. Participants with 25(OH)D levels < 20 ng/ml had a significantly increased risk of metabolic syndrome compared to subjects with levels > 30 ng/ml after adjustment for multiple confounders (OR 2.25, 95% CI 1.25 – 4.07). Plasma 25(OH)D levels were inversely associated with diastolic blood pressure (R = –0.10, p = 0.029) and serum triglyceride levels (R = –0.14, p = 0.002). Conclusion: 25(OH)D deficiency is strongly associated with an increased risk of metabolic syndrome in non-diabetic patients with severe CKD not yet on dialysis, independent of cardiometabolic risk factors and other important regulators of mineral metabolism.

## Introduction 

Chronic kidney disease (CKD) is a growing public health concern, as it is a major risk factor for progression to end stage renal disease, cardiovascular events, and all-cause mortality [1]. Risk-factor modification strategies proven to attenuate risk in the general population are often shown to be non-efficacious in severe CKD patients [2, 3]. The identification of other non-traditional, modifiable cardiovascular (CVD) risk factors in this patient population is critical. 

The metabolic syndrome (MetS), a concurrence of multiple metabolic abnormalities including insulin resistance, hyperglycemia, hypertension, abdominal obesity, elevated serum triglyceride levels and decreased serum high-density lipoprotein cholesterol (HDL-C) levels, is associated with the development of Type 2 diabetes and cardiovascular disease [5]. Concomitant with the epidemic of obesity in the United States (US), the prevalence of MetS is also increasing. In the general adult population, the prevalence is estimated to be 23.7% [5]. Interestingly, the prevalence of MetS increases as kidney function declines [6]. In a study of US adults, the prevalence of MetS was 18% in those with an estimated glomerular filtration rate (eGFR) of ≥ 90 ml/min/1.73 m^2^ and increased to 36.5% in those with an eGFR < 45 ml/min/1.73 m^2^ [6]. Several studies have demonstrated that MetS is a risk factor for death, cardiovascular disease, and incident CKD in the general population [7, 8, 9, 10, 11, 12]. In the CKD population, MetS is also an independent risk factor for cardiovascular events [3, 6, 7]. Modification of traditional risk factors does not ameliorate the risk of cardiovascular disease in CKD patients with MetS [3]. Hence, the identification of other risk factors is paramount. 

25-hydroxyvitamin D (25(OH)D) deficiency has been identified as a nontraditional risk factor for cardiovascular disease in both the general and CKD populations [13, 14]. Vitamin D deficiency is also associated with the development of diabetes [15], insulin resistance [16, 17, 18] and the MetS [19] in the general population. In an analysis of the Third National Health and Nutrition Examination Survey (NHANES), the odds of developing MetS decreased across increasing quintiles of serum 25(OH)D concentrations [19]. Since low serum 25(OH)D levels and the MetS are more common in CKD patients than in the general population, it is reasonable to speculate that 25(OH)D deficiency is associated with MetS. However, to our knowledge, this association has not been examined in patients with severe CKD not requiring dialysis. 

In the present study, we tested the hypothesis that low plasma 25(OH)D level is associated with an increased risk of MetS in patients with severe CKD who participated in the Homocysteinemia in Kidney and End Stage Renal Disease (HOST) Study. 

## Subjects and methods 

### Homocysteinemia in kidney and end stage renal disease study (HOST) 

The details of the HOST Study have been described previously [20]. The HOST Study was a multicenter, prospective, randomized, double-blind, placebo-controlled trial examining the effects of folate, pyridoxine hydrochloride (vitamin B_6_) and cyanocobalamin (vitamin B_12_) on death and cardiovascular events in patients with severe kidney disease and elevated plasma total homocysteine concentrations. Between September 2001 and October 2003, 2,056 participants from 36 veterans affairs medical centers aged 21 years or older with end stage renal disease (ESRD) receiving either maintenance hemodialysis or peritoneal dialysis (n = 751) or estimated creatinine clearance (calculated by the Cockcroft-Gault formula) of less than 30 ml/min but not on chronic dialysis (n = 1,305) and a plasma total homocysteine concentration of 15 µmol/l or higher were enrolled. Participants were excluded if they were pregnant, had a life expectancy less than 6 months, end-stage liver disease or metastatic cancer, taking methotrexate, antifolate medication or anticonvulsants, expected to receive a living related kidney donation in the next 6 months, noncompliant with medications, or unable to give informed consent. All participants provided informed consent and each center’s institutional review board approved the study. Participants were randomized to receive either a once-daily capsule containing 40 mg of folic acid, 100 mg of vitamin B_6_ and 2 mg of vitamin B_12_ or a daily placebo capsule. The primary results showed no significant difference in all-cause mortality or cardiovascular outcomes in the treatment vs. placebo groups [20]. 

### Study population and samples 

Participants who had diabetes (n = 602; 46%) or who initiated chronic hemodialysis within 3 months after randomization (n = 34; 2.6%) or for whom a plasma sample was not available for 25(OH)D measurement (n = 174; 13.2%) were excluded, resulting in a final sample of 495 subjects for the present analysis. The plasma samples were collected 3 months after randomization. The plasma was separated and stored centrally at –80 °C until assays were performed. The HOST executive committee and the cooperative studies program of the department of veterans affairs authorized the use of the stored plasma samples for the measurement of 25(OH)D, 1,25- dihydroxyvitamin D (1,25(OH)_2_D), intact parathyroid hormone (iPTH) and fibroblast growth factor 23 (FGF-23) concentrations. 

### Predictor 

The plasma 25(OH)D level measured 3 months after randomization was the primary exposure variable in this analysis. All laboratory measurements were performed at ARUP Laboratories at the University of Utah. 25(OH)D was measured by a commercial competitive chemiluminescent immunoassay on a Liaison analyzer from DiaSorin, Stillwater, MN, USA. The analytical measurement range for the 25(OH)D assay is 7 – 150 ng/ml. The intra-assay coefficients of variations (CVs) were 5.6% and 4.5% at 11 and 28 ng/ml, respectively. The inter-assay CVs were 9.1% and 5.6% at 16 and 51 ng/ml, respectively. Serum total cholesterol, low-density lipoprotein cholesterol (LDL-C), HDL-C and triglyceride levels were measured at each local site using standard techniques. A modified kinetic Jaffe reaction at each local site was used to measure serum creatinine [21]. 

### Outcome 

MetS was the primary outcome of interest and was defined based on the national cholesterol education program/adult treatment panel (ATP) III guidelines. MetS was defined as the presence of all three of the following components: 1) serum total triglycerides ≥ 150 mg/dl or drug treatment for hypertriglyceridemia; 2) HDL-C < 50 mg/dl for women and < 40 mg/dl for men or drug treatment for dyslipidemia; 3) blood pressure ≥ 130/85 mm Hg or drug treatment for hypertension. Abdominal obesity and fasting plasma glucose were not included in the definition as this information was not available in the HOST database. 

### Baseline demographic and clinical data 

Baseline information collected at the time of randomization included a complete history and physical examination, demographics, health status, smoking status, history of alcohol intake, etiology of kidney disease, history of hypertension and cardiovascular disease and use of medications including antihypertensive agents and lipid-modifying drugs. Systolic and diastolic blood pressure was measured in a standard fashion [20]. Although estimated creatinine clearance (calculated by the Cockcroft-Gault formula) was used for eligibility of the HOST study, for the purpose of this analysis, GFR was estimated using the 4-variable MDRD prediction equation [22]. 

Plasma 1,25(OH)_2_D was measured by a commercial competitive radioimmunoassay from DiaSorin, Stillwater, MN, USA. The range of the assay is 5 – 200 pg/ml. The intra-assay CV was 12.6% and 9.7% at 13 and 45 pg/ml, respectively. The inter-assay CVs were 21.4% and 14.7% at 25 pg/ml and 56 pg/ml, respectively. iPTH was measured by an electrochemiluminescent immunoassay with a reference range of 15 – 65 pg/ml. The intra and interassay CVs were both less than 5%. C-terminal FGF-23 concentrations were measured using a second-generation two-site ELISA kit (Immutopics, San Clemente, CA, USA) with antibodies directed against two epitopes within the C-terminal region of the FGF-23 molecule [23]. The analytical measurement range for the FGF-23 assay was 3.0 – 2,300 RU/ml. The intra-assay coefficients of variations (CVs) were 2.6% and 1.4% at 32.1 and 299.2 RU/ml, respectively. The inter-assay CVs were 3.4% and 4.4% at 32.1 and 299.2 RU/ml, respectively. All these laboratory measurements were performed at ARUP Laboratories at the University of Utah. Serum albumin, serum calcium and serum phosphorus were measured at local sites using standard techniques. 

### Statistical analysis 

Demographic and clinical data including cardiovascular risk factors were compared across 25(OH)D levels through the use of the chi-square test for categorical data, ANOVA for continuous variables that are approximately normally distributed, and the Kruskall-Wallis test for skewed continuous variables. 

Plasma 25(OH)D level was evaluated as a categorical variable. Plasma 25(OH)D levels were divided into clinically meaningful cutoffs based on previous studies: < 20, 20 – 30 and > 30 ng/ml, with > 30 ng/ml as the reference group [14, 24, 25]. The cross-sectional association between 25(OH)D levels and MetS was analyzed using logistic regression models. We considered a priori variables that may be associated with vitamin D concentrations and metabolic syndrome as potential confounders in multivariate models, including important clinical variables, cardiometabolic risk factors, and factors related to calcium and phosphorus metabolism. To permit the most useful interpretation of our results in addition to unadjusted models, we used the following concentrations of adjustment. Model 1 included age, gender, race and season; Model 2 included covariates in Model 1 plus BMI, history of cardiovascular disease, treatment arm, serum albumin levels and eGFR; Model 3 included covariates in Model 2 plus smoking status, alcohol intake, serum calcium, phosphorus, plasma iPTH, 1,25(OH)_2_D, and FGF-23; Model 4 included covariates in Model 3 plus history of hypertension. Two-tailed values of p < 0.05 were considered statistically significant. Pearson correlation coefficients were used to examine the relationship between 25(OH)D levels and components of the MetS including systolic and diastolic blood pressure, triglycerides and HDL-C. The relationship between 25(OH)D levels and BMI was also examined. All statistical analyses were performed with SAS software, version 9.13 (SAS Institute, Cary, NC, USA). 

## Results 

Table 1 lists the baseline characteristics of the participants across plasma 25(OH)D levels. Participants with the lowest vitamin D levels (< 20 ng/ml) were more likely to be younger, Black, to smoke, to have higher total serum total cholesterol, triglyceride and plasma iPTH levels and lower serum albumin and plasma 1,25(OH)_2_D levels. Participants with vitamin D levels < 20 ng/ml had a lower prevalence of cardiovascular disease than those with levels > 30 ng/ml. There was no significant relationship between plasma FGF-23 concentrations and 25(OH)D levels. The percentage of participants with a BMI of < 25, 25 – 30 and > 30 kg/m^2^ was 38.2%, 41.0% and 20.8%, respectively. No difference in the concentrations of mineral metabolism markers (25(OH)D, 1,25(OH)_2_D, FGF-23 or iPTH) were observed based on randomization to either the intervention or the placebo group in the original HOST study. 

**Figure 1.**
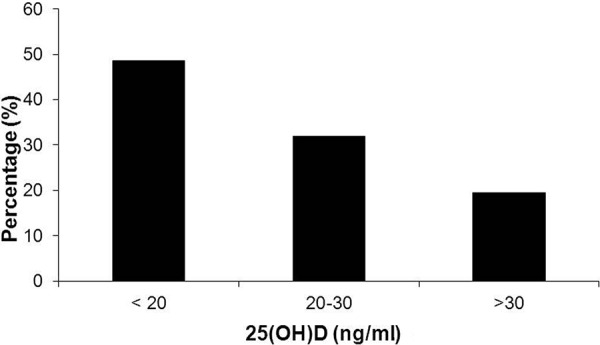
Prevalence of metabolic syndrome across plasma 25-hydroxyvitamin D levels.

The prevalence of MetS across 25(OH)D levels is depicted in Figure 1. The prevalence of MetS increased as 25(OH)D levels declined, with the highest prevalence in patients with 25(OH)D levels < 20 ng/ml (percentage of subjects with MetS: 48.6%, 31.9% and 19.5% for 25(OH)D levels < 20, 20 – 30 and > 30 ng/ml, respectively). 

We evaluated the association between plasma 25(OH)D levels with individual components of the MetS. We observed that higher plasma 25(OH)D levels were inversely correlated with diastolic blood pressure (r = –0.10, p = 0.02), triglycerides (r = –0.14, p = 0.001) but not with low-sex specific cutoff of HDL-C (p > 0.20 for both) and systolic blood pressure (p = 0.89). Even though BMI was not included in our definition of MetS we also examined the correlation between plasma 25(OH)D levels and BMI given the known relationship between obesity and 25(OH)D. We found that 25(OH)D levels were inversely correlated with BMI (r = –0.10, p = 0.03). 

The multivariable-adjusted associations of 25(OH)D levels with MetS are shown in Table 2. After adjustment for age, gender, race and season (Model 1), participants with plasma 25(OH)D levels < 20 ng/ml had an 86% increased odds of MetS compared to participants with levels > 30 ng/ml (OR 1.86, 95% CI 1.11 – 3.07). After further adjustment for BMI, treatment arm, CVD, eGFR and serum albumin levels (Model 2) participants with plasma 25(OH)D levels < 20 ng/ml had over a 2-fold increased risk of MetS compared to those with levels > 30 ng/ml (OR 2.11, 95% CI 1.23-3.63). A plasma 25(OH)D level < 20 ng/ml remained strongly associated with an increased risk of MetS after adjustment for the covariates in Models 1 and 2 plus regulators of mineral metabolism including serum calcium, phosphorus, plasma 1,25(OH)_2_D, iPTH and FGF-23, smoking status and alcohol intake (OR 2.27, 95% CI 1.26 – 4.10). The results were unchanged when hypertension was added in Model 4 (OR 2.25, 95% CI 1.25 – 4.07). Compared to a level > 30 ng/ml, there was no increased risk of MetS in participants with a plasma 25(OH)D level between 20 and 30 ng/ml. Notably, when 25(OH)D levels were subdivided by 1 ng/ml decreases instead of clinically accepted cutoffs of 25(OH)D deficiency, the risk of metabolic syndrome was markedly increased when the 25(OH)D level was < 20 ng/ml. We repeated the models using 1,25(OH)_2_D, iPTH, and FGF-23 concentrations as the exposure variable and found no significant association of these variables with MetS (results not shown). 

## Discussion 

In non-diabetic patients with severe CKD not yet on dialysis in the present study, lower plasma 25(OH)D levels are independently associated with a higher risk of MetS. These findings were present even after adjusting for traditional risk factors for cardiometabolic disorders and for other important regulators of mineral metabolism (calcium, phosphorus, 1,25(OH)_2_D, iPTH and FGF-23). Additionally, plasma 25(OH)D levels were inversely associated with individual components of the MetS. 

Most of our understanding of the association of vitamin D levels and MetS are derived from the general population. Ford et al. [19], used a cross-sectional analysis of the THIRD NATIONAL HEALTH AND NUTRITION EXAMINATION SURVEY (NHANES) database to show that the odds of having MetS decreased across increasing quintiles of 25(OH)D levels. Several other observational studies have confirmed an inverse association between 25(OH)D levels with the risk of MetS in the general population [26, 27, 28, 29, 30], while others have found no such association [31, 32, 33]. The conflicting results in the literature may be due to differences in the study populations, baseline 25(OH)D levels and the inability of some studies to control for adiposity and iPTH. For example, mean 25(OH)D levels were greater than 40 ng/ml in a study of community dwelling older adults [32] that did not find an association between 25(OH)D and MetS, whereas 25(OH)D levels were on average 20 ng/ml in studies that did find such an association [28]. Furthermore, several studies that found an association between lower 25(OH)D levels and MetS did not control for adiposity, iPTH or kidney function. All of these factors are associated with MetS and vitamin D levels and therefore should be considered important confounders [19, 28, 30]. Thus, our findings are of note as we found a significant inverse association between plasma 25(OH)D levels and MetS in patients with CKD, even after controlling for iPTH and BMI. BMI is a measure of the degree of adiposity but may not be a good surrogate for abdominal obesity. Unfortunately we did not have information on waist circumference in our study. Nonetheless, to our knowledge, this is the first study evaluating the cross-sectional relationship between plasma 25(OH)D levels and MetS in patients with severe CKD not yet requiring dialysis. The magnitude of the association between 25(OH)D levels and MetS in our study is similar to studies in the general population. For example, similar to our findings, in the study by Ford et al. [19], non-diabetic subjects in the lowest quintile of 25(OH)D had over a 2-fold increased risk of MetS compared to subjects in the highest quintile (adjusted OR 2.17, p < 0.001). Another study of middle aged and elderly Chinese subjects found that those in the lowest quintile of 25(OH)D had a 52% increased risk of MetS compared to those in the highest quintile (adjusted OR 1.52, p = 0.0002) [30]. However, these findings in the general population may have been attenuated had they been adjusted for iPTH and kidney function. Thus, our ability to adjust for other regulators of mineral metabolism including iPTH, 1,25(OH)_2_D and FGF23 is a key strength to our findings. 

The incidence and prevalence of MetS have been shown to increase as kidney function declines [6, 34]. In patients with CKD, MetS is important for several reasons. First, it is a significant independent predictor of CVD which is the leading cause of death in patients with CKD. In a study of 200 patients with advanced CKD, MetS was independently associated with over a 2-fold increased risk of CVD [3]. In the Multiethnic Study of Atherosclerosis (MESA) subjects with CKD and MetS had an adjusted hazard ratio of 5.56 (95% CI 3.72 – 8.12) for incident cardiovascular disease compared to subjects without MetS [35]. Second, MetS may be a risk factor for progression of CKD. In a study of patients with CKD Stages 1 – 3, patients with MetS had a 60% increased risk of CKD progression compared to those without MetS. However, in patients with more severe CKD (Stage 4 and 5) MetS did not predict progression [36]. Whether MetS results in risk of CKD progression beyond its individual components is still unclear and requires further study. Data does suggest that prevention or early treatment of MetS could potentially improve outcomes in patients with CKD. Our findings suggest that vitamin D deficiency may be a modifiable risk factor for MetS in patients with severe CKD. 

The mechanism by which vitamin D deficiency causes MetS in patients with CKD is likely multifactorial with abnormalities in glucose and insulin metabolism, obesity, hypertension and dyslipidemia all playing a role. Observational studies have noted an association between decreased serum 25(OH)D level and visceral adiposity [37, 38, 39]. 25(OH)D levels progressively decrease as the percent of total body fat increases [40]. It is thought that because vitamin D is a fat-soluble vitamin, it is sequestered in fat stores which represent a barrier for its release into the circulation [37, 38, 39]. However, Bell et al. [41] hypothesized that hypovitaminosis D in obese subjects is the result of secondary hyperparathyroidism and increased serum 1,25(OH)_2_D levels, which provides negative feedback inhibition on liver 25(OH)D synthesis. Thus, adiposity may play a large role in the association between vitamin D and MetS. Information on abdominal obesity was not available in the HOST Study, but we were able to adjust for BMI, plasma PTH and 1,25(OH)_2_D levels and show an association between vitamin D and MetS in CKD patients independent of these important confounders. 

Dyslipidemia is another important component of the MetS. Concordant with studies done in the general population, we found an inverse association between plasma 25(OH)D levels and serum triglyceride levels [6, 27]. The mechanism for this association is not well understood but it has been postulated that low 25(OH)D levels decrease intestinal calcium absorption leading to decreased fecal excretion of cholesterol [42]. Alternatively, 25(OH)D deficiency may result in decreased hepatocellular calcium levels which in turn stimulate hepatic triglyceride formation [43]. Further studies are needed to evaluate the relationship of low 25(OH)D levels with high triglycerides levels. 

Another way 25(OH)D may be contributing to MetS in patients with CKD is through the development of hypertension. Several studies have shown an association between vitamin D and hypertension in the general population [6, 44]. In our cohort of CKD patients, we found an inverse correlation between low plasma 25(OH)D levels and higher diastolic blood pressures. The exact mechanism by which vitamin D deficiency results in hypertension is unknown but there are several postulated mechanisms including upregulation of the renin-angiotensin system, decreased production of nitric oxide by endothelial cells, and an increase in plasminogen activator inhibitor-1 [6, 44]. Small studies in the general population have shown decreases in blood pressure following supplementation with vitamin D [45]. However, to date, no randomized trials evaluating the effect of vitamin D supplementation on hypertension have been performed in the CKD population. 

Although we did not have data on insulin resistance in the HOST study, abnormalities in glucose and insulin metabolism likely play a role in the association between vitamin D and MetS in patients with CKD. Several studies in the general population have found that patients with impaired glucose metabolism or diabetes have lower serum 25(OH)D levels than subjects with normal glucose tolerance [48]. Serum 25(OH)D levels have also been found to be inversely associated with plasma glucose, insulin resistance and β cell function in several observational studies [15, 17, 28, 49]. 

Insulin resistance increases as kidney function declines but the mechanism behind this finding is unclear [17]. Given that the prevalence of 25(OH)D deficiency also increases as kidney function declines, it is tempting to speculate that 25(OH)D deficiency is an important cause of the increased insulin resistance in patients with CKD. Randomized trials are needed to evaluate whether supplementation with vitamin D improves insulin resistance and decreases the risk of MetS in patients with CKD. 

Principal limitations to this study include those that are inherent in cross-sectional analysis, such as the inability to establish a causal or temporal relationship between 25(OH)D and MetS. Second, the patient population is predominantly male thereby making these findings less generalizable to female patients with CKD. Third, data on abdominal obesity and hyperglycemia, the other components of metabolic syndrome, were not available in the HOST database for our analysis. This exclusion of abdominal obesity is important because adiposity is associated with low serum 25(OH)D levels in the general population. Studies have shown that in CKD Stage 3 – 4, insulin resistance is more dependent on BMI than on kidney function [52]. Even though we were not able to control for abdominal obesity, we were able to adjust for BMI, 1,25(OH)_2_D, and iPTH all of which are important confounders in the relationship between vitamin D and MetS. Fourth, we did not have information on patient use of nutritional vitamin D supplements or active vitamin D analogs. However, given the time period the HOST study was performed (September 2001 through October 2003), active vitamin D analogues and nutritional vitamin D supplements were not likely routinely given in patients with CKD not requiring dialysis as KDOQI guidelines were not available until the end of 2002. Further, regardless of the use of nutritional vitamin D supplementation, the end result was a significant association between low plasma 25(OH)D levels and the presence of MetS. Additionally, we did not have any information on socioeconomic status, diet, or history of liver disease all of which potentially can affect 25(OH)D levels. Finally, the definition of kidney disease was based upon estimated MDRD-GFR rather than more precise measures of kidney function such as iothalamate clearance. 

Despite these limitations, our study has several important strengths. First, to our knowledge this is the first study examining the association of 25(OH)D levels with MetS in patients with severe CKD not requiring dialysis. Second, the HOST study from which these data were obtained had a large patient population and a comprehensive dataset allowing for adjustment of important cardiometabolic risk factors and regulators of mineral metabolism. Third, all laboratory measurements including 25(OH)D, 1,25(OH)_2_D, PTH, FGF-23, triglycerides and HDL-C were performed in a standardized fashion. 

In conclusion, 25(OH)D deficiency is strongly associated with an increased risk of MetS in non-diabetic patients with severe CKD, independent of cardiometabolic disorders, kidney function and other important regulators of mineral metabolism. These data suggest that low plasma 25(OH)D levels may be a modifiable risk factor for MetS in patients with CKD and supplementation may result in improved morbidity and mortality. A prospective, randomized trial should be performed examining the effect of nutritional vitamin D supplementation on the development of MetS and components of the MetS in patients with CKD. 

## Acknowledgments 

The research reported in this study was supported by the Department of Veterans Affairs Cooperative Studies Program and the HOST Executive Committee (Rex L. Jamison, Pamela Hartigan, James Kaufman, David S. Goldfarb, Stuart R. Warren, Peter D. Guarino, and J. Michael Gaziano). A full list of Veterans Affairs HOST Site Investigators and institutions can be found at http://www.ncbi.nl.gov/pubmed/17848650. Additional support came from the National Institute of Diabetes and Digestive and Kidney Disease grant K23DK087859-01A1, R01 DK081473 and R01 DK078112, an AMGEN fellowship grant and an Abbot and Genzyme independent investigator grant. 

**Table 1.** Baseline characteristics of participants by plasma 25(OH)D levels.

Characteristic	Plasma 25(OH)D			p value for trend
	< 20 ng/ml (n = 228)	20 – 30 ng/ml (n = 162)	> 30 ng/ml (n = 105)	
Age (years)	68 ± 12	71 ± 12	72 ± 10	0.0016
Sex (% Male)	98.2	98.8	99.0	0.54
Race (% Black)	39.5	18.2	14.6	< 0.0001
CVD (%)	44.7	50.6	61.9	0.004
Hypertension (%)	95.6	95.7	93.3	0.43
Current smoking (%)	62.1	42.7	39.1	0.004
Alcoholic drinks per week	1.30 ± 3.6	1.50 ± 5.3	0.73 ± 2.2	0.34
Treatment arm of HOST (%)	49.6	46.9	52.4	0.76
BMI (kg/m^2^)	26.8 ± 4.3	26.6 ± 4.8	26.0 ± 4.0	0.14
SBP (mmHg)	140 ± 22	140 ± 21	138 ± 18	0.53
DBP (mmHg)	75 ± 13	73 ± 13	72 ± 11	0.059
MDRD-eGFR (ml/min/1.73 m^2^)	17.7 ± 6.6	17.9 ± 6.6	19.4 ± 6.7	0.04
Albumin (g/dl)	4.1 ± 0.4	4.2 ± 0.4	4.2 ± 0.5	0.0006
Phosphorus (mg/dl)	4.2 ± 1.2	4.3 ± 2.1	4.1 ± 1.0	0.68
Calcium (mg/dl)	8.9 ± 0.7	8.9 ± 0.7	9.0 ± 0.6	0.27
1,25(OH)_2_D (pg/ml)	17.7 ± 8.1	24.2 ± 12.6	27.7 ± 12.4	< 0.0001
iPTH (pg/ml)	225 ± 180	158 ± 120	144 ± 123	< 0.0001
FGF-23 (RU/ml)	1,212 ± 3,050	793 ± 1,024	873 ± 2,252	0.14
Total cholesterol (mg/dl)	181 ± 43	176 ± 38	164 ± 37	0.0006
Triglycerides (mg/dl)	197 ± 180	169 ± 95	142 ± 71	0.0006
LDL-C (mg/dl)	100 ± 33	99 ± 33	93 ± 31	0.13
HDL-C (mg/dl)	45 ± 19	44 ± 16	43 ± 11	0.32

Values are expressed as mean ± standard deviation unless otherwise specified. CVD = cardiovascular disease; BMI = body mass index; SBP = systolic blood pressure; DBP = diastolic blood pressure; MDRD-GFR = glomerular filtration rate calculated using the Modification of Diet in Renal Disease formula; TG = triglyceride; LDL = low density lipoprotein cholesterol; HDL = high density lipoprotein cholesterol; Calcium = albumin-adjusted serum calcium; 25(OH)D = 25-hydroxyvitamin D; 1,25(OH)_2_D = 1,25-dihydroxyvitamin D; FGF-23 = fibroblast growth factor 23; iPTH = intact parathyroid hormone.

**Table 2.** Odds ratio (95% CI) of metabolic syndrome according to plasma 25-hydroxyvitamin D levels.

	Plasma 25(OH)D		
	< 20 ng/ml	20 – 30 ng/ml	> 30 ng/ml
Model 1	1.86 (1.11, 3.07)	1.21 (0.73, 2.02)	1.00 (REF)
Model 2	2.11 (1.23, 3.63)	1.33 (0.78, 2.27)	1.00 (REF)
Model 3	2.27 (1.26, 4.10)	1.34 (0.78, 2.31)	1.00 (REF)
Model 4	2.25 (1.25, 4.07)	1.34 (0.78, 2.30)	1.00 (REF)

Model 1: adjusted for age, gender, race and season; Model 2: adjusted for covariates in model 1 plus body mass index, treatment arm, history of cardiovascular disease, estimated glomerular filtration rate and serum albumin levels; Model 3: adjusted for covariates in model 2 plus smoking status, alcohol intake, serum calcium, phosphorus, plasma intact parathyroid hormone, fibroblast growth factor 23, and 1,25-dihydroxyvitamin D; Model 4: adjusted for covariates in model 3 plus hypertension.
